# Macular thickness measurements of healthy, naïve cynomolgus monkeys assessed with spectral-domain optical coherence tomography (SD-OCT)

**DOI:** 10.1371/journal.pone.0222850

**Published:** 2019-10-07

**Authors:** Nora Denk, Peter Maloca, Guido Steiner, Christian Freichel, Simon Bassett, Tobias K. Schnitzer, Pascal W. Hasler

**Affiliations:** 1 Pharma Research and Early Development (pRED), Pharmaceutical Sciences (PS), Roche Innovation Center Basel, Basel, Switzerland; 2 OCTlab Research Laboratory, Department of Ophthalmology, University of Basel, Basel, Switzerland; 3 Moorfields Eye Hospital, London, United Kingdom; 4 Institute of Molecular and Clinical Ophthalmology Basel (IOB), Basel, Switzerland; University of Miami Bascom Palmer Eye Institute, UNITED STATES

## Abstract

The purpose of this study was to measure central macular thickness in an unprecedented number of cynomolgus monkeys. Macular thickness was measured with Heidelberg spectral-domain OCT in 320 eyes of healthy and treatment-naïve cynomolgus monkeys (80 males and 80 females). The macula was successfully measured in all 320 eyes. Macular thickness was not significantly different between the sexes. The mean central macular thickness was 244 μm (+/- 21 μm). Macular thicknesses in the quadrants were 327 +/-17 μm (temporal inner), 339 +/- 17 μm (inferior inner), 341 +/- 14 μm (superior inner), 341 +/-18 μm (nasal inner), and 299 +/- 20 μm (temporal outer), 320 +/- 16 μm (superior outer), 332 +/-23 μm (inferior outer), and 337 +/-18 μm (nasal outer). Highly significant differences between the nasal and temporal quadrants were detected. This study successfully demonstrated the feasibility of retinal thickness measurements in healthy cynomolgus monkeys. The present findings indicate that the macula is thicker in cynomolgus monkeys than in humans and provide important normative data for future studies.

## Introduction

Macular thickness and morphology have served as successful biomarkers in the treatment of macular edema in various diseases, such as age-related macular degeneration (AMD), uveitis or retinal vein occlusion [1–3]. In contrast, thinning of the central retina can occur due to retinal degeneration and atrophy [4]. Therefore, understanding the range of normal macular thickness values in nonhuman primates used in toxicology studies is important to distinguish treatment-related changes from normal variation.

Optical coherence tomography (OCT) is a commonly used technique to facilitate longitudinal noninvasive assessment of retinal structures during the time course of preclinical studies. OCT is a noninvasive, high-resolution, in vivo imaging technique that provides a real-time cross-sectional images of ocular structures and is most commonly used to evaluate the retina and optic nerve at an axial resolution of 3.9 to 7 μm. Cross-sectional images of the retina with the quality of a low power microscope can be obtained in vivo by OCT measurements, and this technique is widely used in humans. The use of OCT in laboratory animals has been described in a variety of species and is increasingly employed in animal models of human disease and preclinical trials [5–9].

Many studies have reported normative data for macular thickness in humans using various commercially available standard domain optical coherence tomography (SD-OCT) devices [4, 10–12]. Values vary by population subsets and instruments used for analysis [13]. Automated differentiation of vitreoretinal and retinochoroidal differentiation has been developed for use in humans but is less accurate in nonhuman primates, despite the close similarity of their ocular structures. Therefore, accurate thickness measurements in these species require manual, time-consuming correction. Consequently, these measurements are not routinely employed in large data sets. Therefore, the purpose of this study was to measure and define normative data for macular thickness in an unprecedented number of subjects for the first time.

## Materials and methods

### Animals and husbandry

The review and assessment of data in this study was accomplished via retrospective analysis of raw data from studies conducted in the routine support of pharmaceutical product development. Therefore, no additional animals were used to acquire these data. The OCT images reviewed in the current study were part of safety assessment studies, and animals were consecutively followed. These primary studies have been approved by the Institutional Animal Care and Use Committees (IACUC) of the respective institutions. Studies were approved by one of the following IACUCs depending on the study: Charles River Laboratories Montreal, ULC Institutional Animal Care and Use Committee (CR-MTL IACUC), IACUC Charles River Laboratories Reno (OLAW Assurance No. D16-00594) and Institutional Animal Care and Use Committee (Covance Laboratories Inc., Madison, WI) (OLAW Assurance #D16-00137 (A3218-01). During the study, the care and use of animals was conducted according to the guidelines of the US National Research Council or the Canadian Council on Animal Care.

Eighty female and 80 male cynomolgus monkeys of Mauritian genetic background who were between 30 and 50 months of age and weighed between 2.5 and 5.5 kg were used in these studies. Animals were group housed in stainless steel cages, according to European housing standards described in Annex III of Directive 2010/63/EU. The temperature of the animal room was maintained between 20°C and 26°C, and the humidity ranged between 30% and 70%. The light cycle was 12 hours of light and 12 hours of darkness, except during designated procedures. Animals were fed PMI Nutrition International Certified Primate Chow. Municipal tap water treated by reverse osmosis and ultraviolet irradiation was freely available to each animal via an automatic watering system. Psychological and environmental enrichment was provided to animals except during study procedures and activities. All animals included in the present study tested negative for Tb.

### Optical coherence tomography imaging and evaluation

Scanning laser ophthalmoscopy (SLO) was obtained simultaneously with SD-OCT using the Spectralis HRA +OCT Heidelberg platform (Heidelberg Engineering, Heidelberg, Germany). Imaging of the animals was performed under general anesthesia to minimize stress for the animals and ensure a stable eye position. Animals were anesthetized with a mixture of ketamine 10 mg/kg and dexmedetomidine 25 μg/kg IM. Immediately before the start of the OCT imaging, a single dose of midazolam 0.2 mg/kg IM was administered to keep the eyes centrally positioned. Exclusion criteria for scan selection were pathological background findings, such as corneal opacities or cataracts, as well as a scan quality of less than 25.

**Fig 1:**
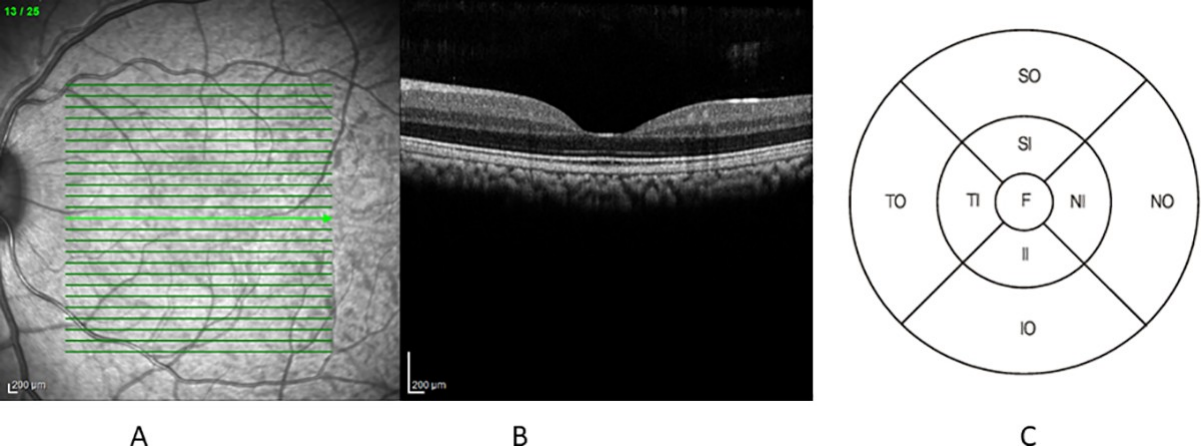
Example of macular thickness measurements obtained using an SD-OCT. (A) Scanning laser ophthalmoscopy (SLO) image of the fundus. The green lines indicate the individual OCT scan lines. (B) OCT scan representing the bright green error on the SLO image. Red lines illustrate the segmentation lines. (C) Depiction of the standard Early Treatment of Diabetic Retinopathy Study (ETDRS) map. Map centers on the thinnest point of the fovea (O–Outer, I–Inner, S–superior, I–inferior. N–nasal, T–temporal, F–foveal).

The OCT volume scans were performed through the dilated pupil (topical tropicamide treatment) on cubes with 25 to 31 raster lines, separated by 120 to 218 μm depending on the study protocol. The high-resolution setting was used, and at least 30 frames were averaged for each B scan. Each scan was individually reviewed retrospectively, and segmentation lines of the volume scans were adjusted manually if necessary to ensure accuracy in macular thickness measurements. Retinal thickness was defined as the distance between the vitreoretinal interface and the inner border of the retinal pigment epithelium (RPE) (Fig 1) as described previously. [13, 14].

Macular thickness was reported in a modified Early Treatment of Diabetic Retinopathy Study (ETDRS) macular map commonly used in humans where the central subfield was 1 mm in diameter and the inner and outer circular subfields had diameters of 3 and 6 mm, respectively. The zones (fovea, inner, and outer macula) were further divided by the grid into superior, inferior, nasal, and temporal regions (Fig 1). Each ETDRS grid was manually repositioned with the center matching the thinnest point of the macula. The retinal thickness at this point was defined as the central foveal thickness. OCT scans were individually reviewed to ensure that the quality was sufficient to allow for data interpretation, i.e., the focus of the scan allowed for the determination of the RPE border.

### Statistical analysis

Graphical exploration and consistency checking preceded statistical summarization and analysis of data. As a result, the complete set of retinal thickness measurements from our balanced data set of 80 male and 80 female monkeys (320 eyes in total) were included in our assessments.

Descriptive statistics (means, standard deviations, quantiles) were calculated using the corresponding base functions of R (version 3.4.0) [15]. Unless stated otherwise, standard deviations are reported as variability measures around the mean to properly reflect the spread in the population. Kernel density estimates of the thickness distribution were obtained from R’s density function with default heuristics for the selection of the smoothing bandwidth. Homogeneity of variances was assessed with the Fligner-Killeen test for median-centered, not necessarily normally distributed, data.

For statistical inference, the data were modeled with a linear mixed-effect model, including the full ‘eye region * animal sex * lateral side’ interaction as a fixed effect and a nested ‘eye within animal’ random intercept. Heteroscedasticity was accounted for by allowing different variances for each level of the ‘eye region’ and ‘animal sex’ factor combinations. The R modules nlme and emmeans were used to fit the model and obtain relevant estimates or contrasts. A variance component model (nesting the factors eye region, animal, and lateral side) was used to obtain separate estimates for biological (interanimal) variability and intraanimal (left to right eye) variability.

## Results

**Fig 2:**
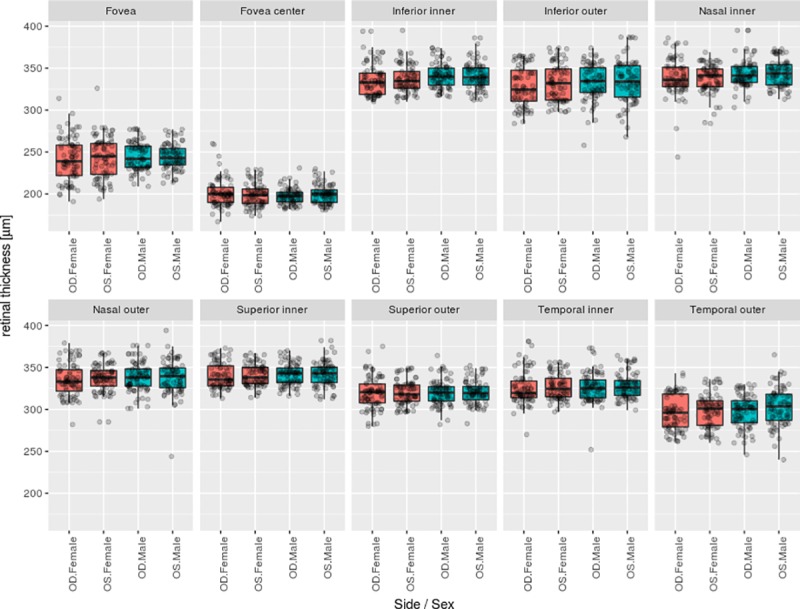
Boxplot representation of the complete retinal thickness data set from 160 cynomolgus monkeys, overlaid with individual measurements. Data is split by lateral side as well as animal sex (red fill: females, blue fill: males).

All 320 eyes from 80 female and 80 male cynomolgus monkeys could be evaluated, and no ocular pathologies were noted in any of the eyes. Furthermore, all eyes matched the inclusion criteria with respect to image quality as described above. Plotting the single values for each area (Fig 2) illustrates a homogenous data set with only a few outlying observations.

**Fig 3:**
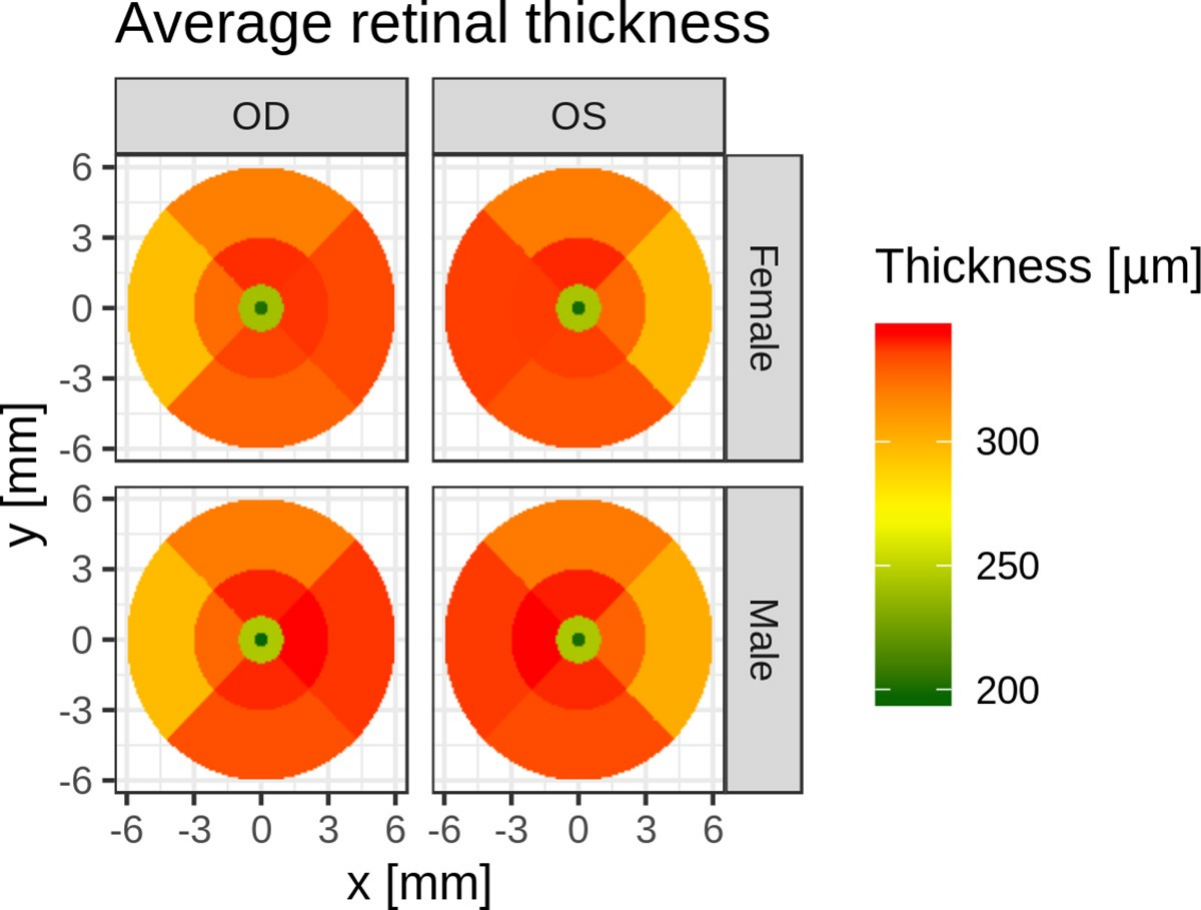
Distribution of retinal thickness values (kernel density estimates) from the entire data set. Panels on the left allow direct comparison between male and female monkeys, while panels on the right side compare the two lateral sides. OS = *lat*.: *oculus sinister* = left eye; OD = *lat*.: *oculus dexter* = right eye.

The overall retinal thickness of the macular volume scans was 320 +/- 18 μm. The mean retinal thickness in males was 321 +/- 17 μm, and in females, it was 318 +/- 18 μm. Mean retinal thickness measurements and their variability are shown in Fig 3 and S1 Table, separately for the sector, lateral side, and sex of the animal. As expected, macular thickness was thinnest in the fovea. The average nasal quadrant was slightly thicker than the average temporal quadrant.

**Fig 4:**
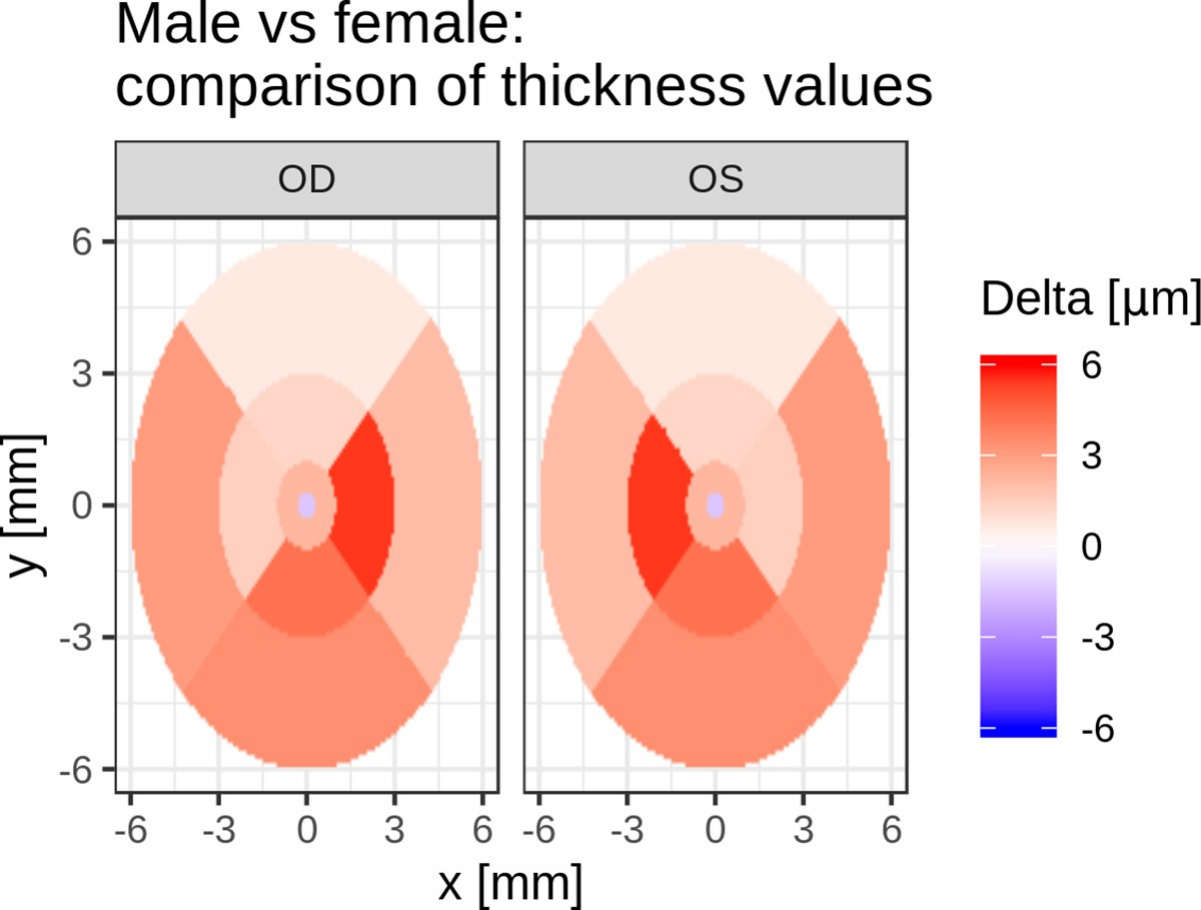
Graphical illustration comparing thickness values in males and females. Thickness differences (male–female) projected on the ETDRS circle. Red shading (positive delta) indicates higher thickness for males than for females. For this illustration, thickness differences were averaged between the left and right eyes before mapping to the ambilateral grid; hence, the plot is perfectly symmetrical.

Overall, males showed slightly thicker maculae than females did (+2.25 μm averaged across regions; p = 0.244), with the exception of the foveal center, which was slightly thicker in females. However, no significance was attributed to this difference globally or for any specific eye region (after correction for multiplicity of testing) (Fig 4). Similarly, no significant difference in average thickness was found in any eye region when comparing values between the eyes.

However, highly significant differences between the nasal and temporal quadrants were present. Retinal thicknesses were 14 μm [95% confidence interval (CI), 12–16 μm, p<0.001] and 38 μm (95% CI, 36–41 μm, p<0.001) greater for the nasal quadrant for the inner and outer sectors, respectively.

The variability in thickness depended on the eye region but was comparable between the two lateral sides (OS versus OD) as well as between males and females. However, the fovea was a notable exception, as we found a slightly larger spread of values across the female monkeys compared with those across the males, given comparable means. This finding was true for both lateral sides. Using the Fligner-Killeen test for heterogeneity of variance, we established significance for these findings (p = 0.048 for OD and p = 0.00068 for OS) even after stringent correction for multiple testing across all 40 comparisons as shown in Fig 4.

The standard deviation of thickness readouts included in S1 Table was calculated for individual eye regions and for OS and OD separately. Thus, these values reflect the between-subject variability of retinal thickness for the respective part of the data. This information is important to note because, as we expect less deviation for the two eyes of one specific monkey, we should not mix different sources of variability by putting all data together and calculating a joint standard deviation. A variance component model showed that the mean standard deviation was 17.9 μm across the eyes of different subjects (averaged across-eye region) but was only 11.6 μm within subjects.

## Discussion

Macular thickness is a key landmark in the evaluation and monitoring of different retinal diseases in humans and nonhuman primates [16, 17].

To our knowledge, this study is the first to report macular thickness data assessed with OCT in cynomolgus monkeys, a species commonly used as animal models of human disease as well as for safety assessment in preclinical trials. This study showed that the mean central thickness of the macula was approximately 26% thicker in monkeys than in humans [12]. There was no significant difference between the left and right eyes or between the sexes. However, as in humans, highly significant differences between the nasal and temporal quadrants were noted. Retinal thicknesses were 14 μm (95% CI, 12–16 μm) and 38 μm (95% CI, 36–41 μm) greater for the nasal quadrant for inner and outer sectors, respectively.

In general, the observed macular thickness was thinnest at the fovea and increased overall toward the parafoveal area, which is consistent with the normal anatomical contour. As in humans, the nasal quadrant was slightly thicker than other quadrants of the 6-mm radius, which is considered to reflect the anatomical relationship of the converging retinal nerve fibers with the optic disc [18] Overall, the macula in cynomolgus monkeys is thicker than all values reported thus far in humans [10–12, 19, 20] However, the thickness profile, i.e., the proportion of thickness of certain subfields of the ETDRS circle closely resembles that of humans, as reported by Nigam *et al*. [11], reflecting an overall high degree of concordance between human and nonhuman primate ocular anatomy.

In humans, an influence of ethnicity on macular thickness has been reported [19, 21–25]. Inner macular thickness and volume are significantly thinner in black and Asian populations than in Caucasian populations. The age and weight of humans also influence macular thickness [11, 14]. Our data set consists of a very uniform population with regard to those parameters. However, further studies are needed to determine whether genetic background (e.g., cynomolgus monkeys of Mauritian versus Asian background), body weight, and age might influence macular thickness. Nonetheless, our population represents the current standard used in preclinical toxicology studies.

Significant differences in macular thickness between men and women in most macular subfields have also been reported, with men having a greater thickness in the central and inner 3-mm zone [26]. Other studies did not observe significant differences in macular thickness between men and women [11], whereas others noted significant differences in certain subfields of the ETDRS circle [19, 27]. Although thickness values were not significantly different between male and female monkeys in this study, we found significantly greater variation in thickness values in the fovea in females than in males. This finding may reflect greater variability in foveal morphology. Again, follow-up studies are needed to further investigate this topic by 3D reconstruction of the foveal pit and comparison by sex.

The strengths of this study include the large sample size and the homogenous composition of the group. A significant shortcoming of our study, as described above, is the current need to manually correct errors in automated segmentation by Spectralis software, which increases the potential possibility of subjective or less accurate readouts. Furthermore, this process was time consuming, precluding the assessment of this readout parameter in large data sets as a routine diagnostic procedure.

In our opinion, the development of segmentation software for the most commonly used species is an important next step toward the objective assessment of OCT scans obtained in toxicology studies and will increase the level of information obtained from those studies (refinement). Ultimately, an ideal setup would allow for the assessment of macular thickness by artificial intelligence programs, paving the way toward innovative, reliable, high-throughput, and maximally informative assessment of OCT data sets. Until then, this retrospective study provides normative data on macular thickness measurement that can be used as reference data in future preclinical studies.

## Supporting information

S1 Table Retinal thickness in μm of the various subgroups (different fields of the ETDRS circle for each gender and each eye (left eye (OS) versus right eye (OD)). The Q values refer to various quantiles. For example, Q10 denotes the 10^th^ percentile of the distribution, and Q50 is equivalent to the median.
(DOCX)
