# Supplementary figures and images for: Macular thickness measurements of healthy, naïve cynomolgus monkeys assessed with spectral-domain optical coherence tomography (SD-OCT)
Source: PLoS One. 2019 Oct 7;14(10):e0222850. doi: 10.1371/journal.pone.0222850 (PMC6779255; doi:10.1371/journal.pone.0222850)

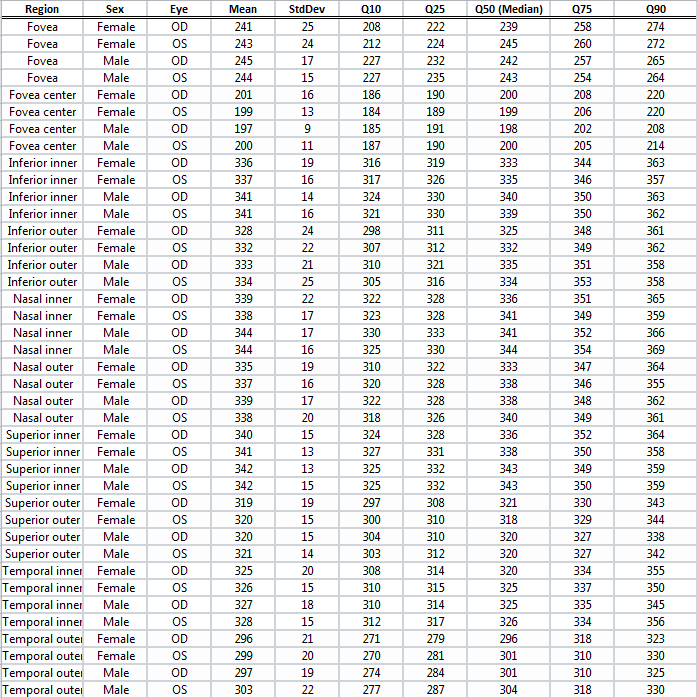

Supplement: S1 Table — The Q values refer to various quantiles. For example, Q10 denotes the 10th percentile of the distribution, and Q50 is equivalent to the median. (DOCX) [file pone.0222850.s001.docx]
